# Supplementary material for: Aptamer Efficacies for In Vitro and In Vivo Modulation of αC-Conotoxin PrXA Pharmacology
Source: Molecules. 2019 Jan 9;24(2):229. doi: 10.3390/molecules24020229 (PMC6359527; doi:10.3390/molecules24020229)
Supplement: Supplementary file 1 [file molecules-24-00229-s001.pdf]

Supplementary information for manuscript: "Aptamer efficacies for *in vitro* and *in vivo* modulation of αC-conotoxin PrXA pharmacology"

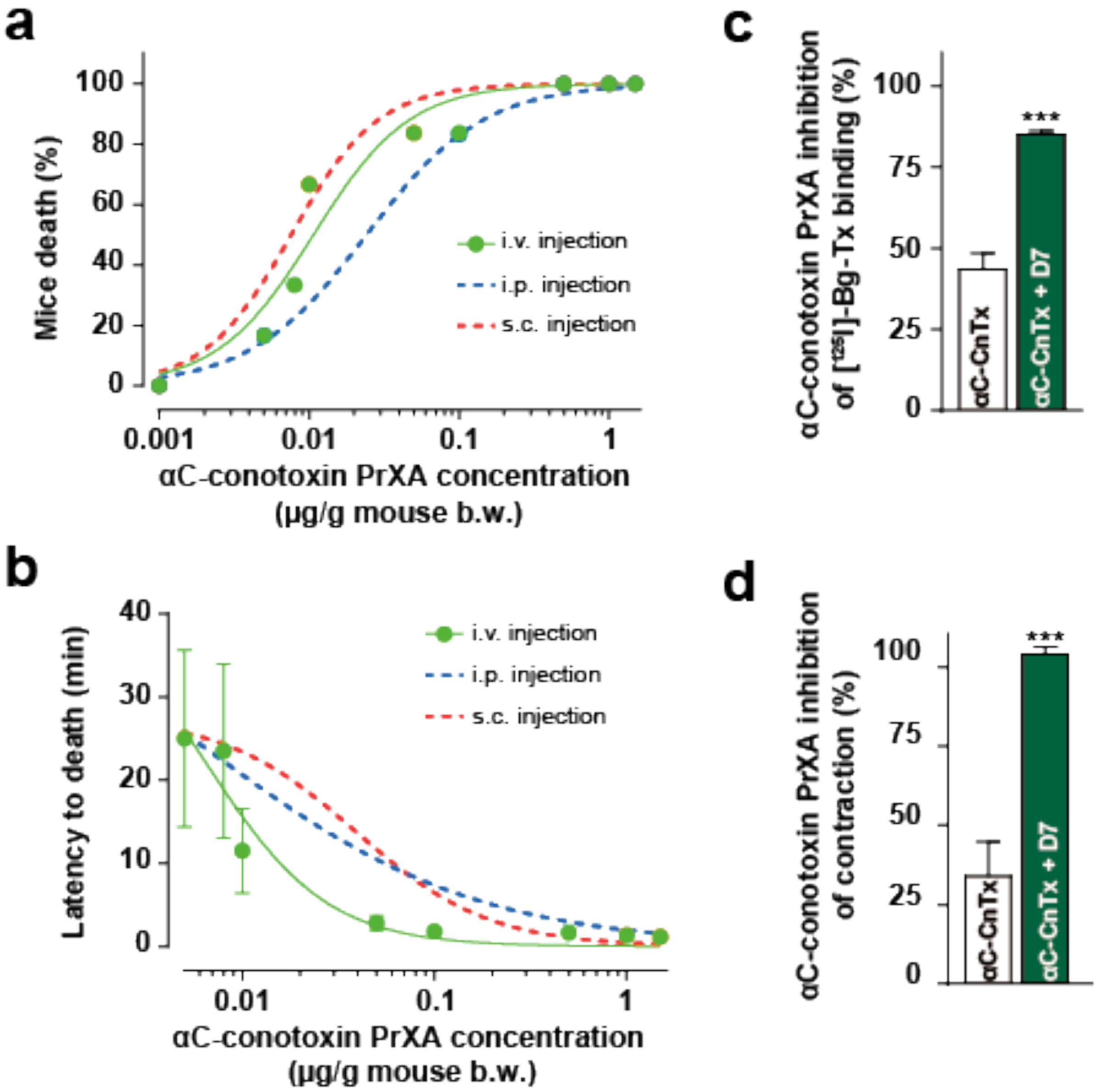

Figure S1. αC-conotoxin PrXA-induced lethality. (**a**) Dose-response curve illustrating the percentage of αC-conotoxin PrXA-mediated mice death after intravenous injection. N=6 mice for each dose. Dotted curves are used for comparison [7]. (**b**) Shortening of latency to death as a function of αC-conotoxin PrXA concentration. N=6 mice for each concentration. (**c**) Effect of the D7 aptamer (green) on 1 µM αC-conotoxin PrXA-mediated inhibition of 1 nM [^{125}I]-α-BgTx binding . (**d**) Effect of the D7 aptamer (green) on 0.03 µM αC-conotoxin PrXA-mediated inhibition of mouse hemidiaphragm muscle contraction.

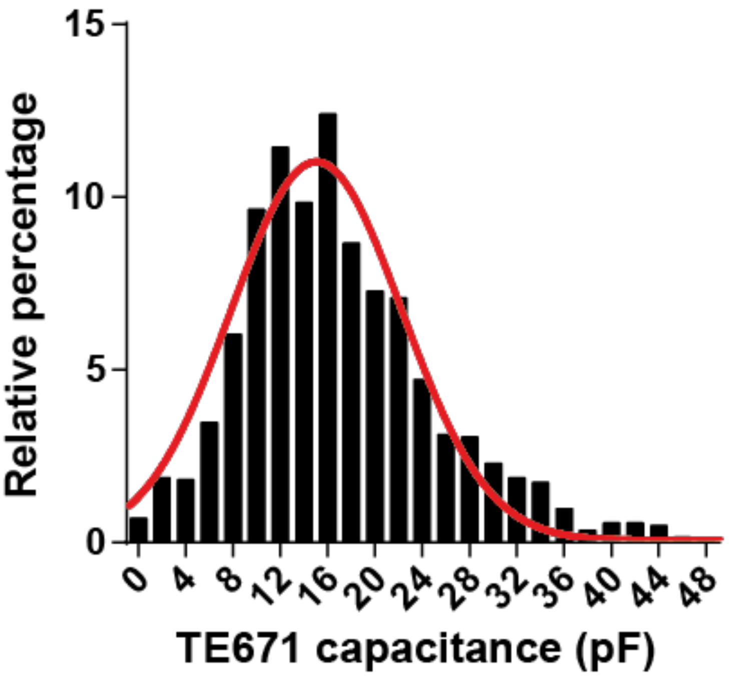

Figure S2. Histogram distribution of TE671 cell capacitance. N=1,444 TE671 cells were included in this study. The histogram was fitted by a Gaussian distribution (Y=Amplitude*exp(-0.5*((X-Mean)/SD)^2)) and yielded the folowing parameters: Mean = 15.19 pF , SD = 6.95 pF . The capacitance interval for each bar equalled 2 pF.

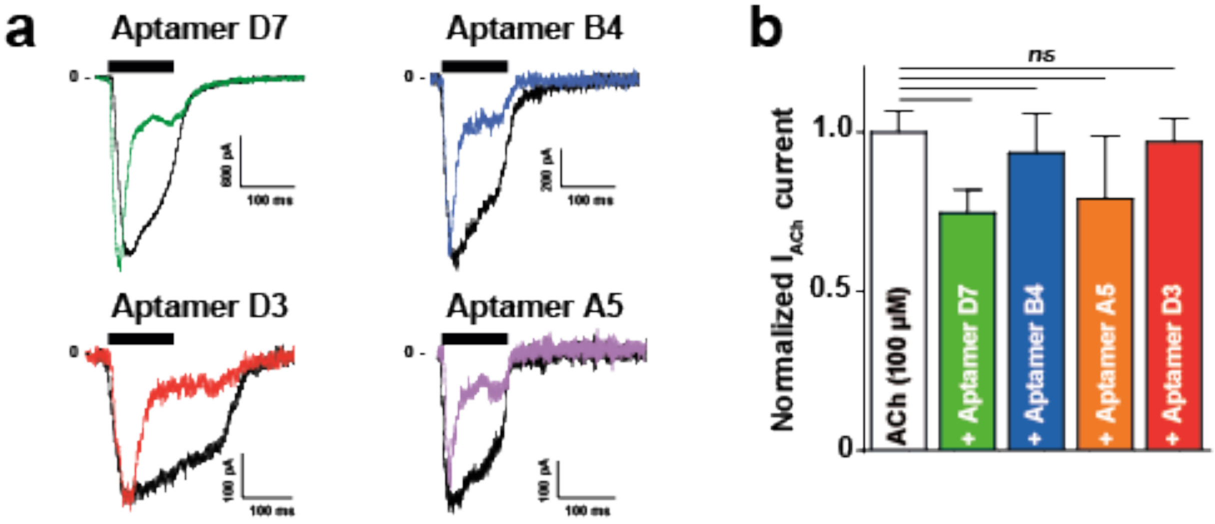

Figure S3. Lack of effect of the aptamers on the ACh response in TE671 cells. (**a**) Representative inward currents mediated by two applications of 100 µM ACh. The first application occurs after a 20-min preincubation of the cells in the presence of 300 nM aptamer (colored trace), while the second application occurs after thorough washout of the aptamer (black trace). The amplitude of the responses were preserved, while less desensitization is observed for the second application as in control conditions. (**b**) First ACh application current densities normalized with regard to the second ACh application current densities in control condition (n=30) and for each aptamer (n=3-17 per aptamer condition).
